# Restoration of elbow flexion in patient with prolonged incomplete brachial plexus injury treated with bipolar latissimus dorsi muscle transfer: A case report

**DOI:** 10.1016/j.ijscr.2024.110554

**Published:** 2024-11-05

**Authors:** R. Malik, Y.S. Robiady, A.R. Ichwan

**Affiliations:** Faculty of Medicine, Department of Orthopaedic Surgery, Universitas Padjadjaran, Bandung, Indonesia

**Keywords:** Brachial plexus injury, Bipolar latissimus dorsi muscle flap

## Abstract

**Introduction:**

The functional deficits resulting from traumatic brachial plexus damage are substantial. In this study, we provide a clinical case with the successful restoration of elbow and shoulder function following a bipolar latissimus dorsi muscle transfer procedure.

**Case presentation:**

A male patient, aged 26, presented at the outpatient clinic with a primary concern of left upper limb weakness that had persisted for a duration of 1.5 years subsequent to an industrial incident. The patient's motor function was assessed as M0 for shoulder abduction and elbow flexion, indicating a range of motion of zero degrees for both movements. Additionally, the Mayo Elbow Score (MES) measurement was recorded as 60. Persistent tingling feelings were observed in the wrist and hand. The neurological assessment revealed a partial impairment of motor function in the radial, median, and ulnar nerves.

**Clinical discussion:**

The electromyographic assessment provided evidence and substantiated the diagnosis of an incomplete left brachial plexopathy. Prompt restoration of elbow flexion was observed on the initial day subsequent to the surgery, accompanied by a minor degree of shoulder abduction. At the six-month mark, the patient demonstrates the ability to execute a 100-degree flexion of the elbow and a 30-degree abduction of the shoulder, exhibiting motor strength at the M4 level.

**Conclusion:**

Following a late brachial plexus injury, the utilization of a bipolar latissimus dorsi muscle flap has demonstrated an exceptional outcome in the context of elbow reconstruction. The preoperative evaluation of donor muscle strength will serve as a reliable indicator for predicting a favorable postoperative result.

## Highlights

• Prompt restoration of elbow flexion was observed on the initial day after the surgery, accompanied by a minor degree of shoulder abduction.
• At the six-month mark, the patient demonstrates the ability to execute a 100-degree flexion of the elbow and a 30-degree abduction of the shoulder, exhibiting motor strength at the M4 level.

## 1 Background

Traumatic brachial plexus injury is an injury involving the network of nerves originating from C5 to T1, which innervates the sensory and motor functions of the shoulder and upper limbs [1]. It is most commonly occurs in active adult males, involved in traffic accidents. The impact on patient's physical and mental health are devastating, due to its significant functional impairment of the patient [2]. The goals of the treatment of brachial plexus injury are including hand reanimation, protective hand sensation, shoulder stability, and elbow function as the first priority in upper extremity reconstruction [3].

Several procedures have been described to restore elbow flexion, including unipolar or bipolar transfer of the pectoralis major, Steindler flexorplasty, unipolar or bipolar transfer of latissimus dorsi, triceps to biceps transfer, pectoralis minor transfer, and sternocleidomastoid transfer. Among some methods has been performed, bipolar latissimus dorsi muscle transfer is a reliable method to restore functional elbow flexion regarding range of motion (>90^0^ elbow flexion) and strength (at least antigravity strength, ≥M3) with acceptable donor morbidity and complication rate. The transfer can be either unipolar or bipolar [4].

Stretching from the dorsal and lumbosacral regions to the humeral shaft, the latissimus dorsi muscle is a broad, flat muscle. With the axilla serving as the apex and the spine as the base, it has a triangle shape. The thoraco dorsal artery, which splits from the subscapular artery that originates from the distal axillary artery, provides its blood supply. The root C5, C6, and C7 of the thoracodorsal nerve innervates the muscle. It is responsible for arm adduction, internal rotation, and retropulsion [5]. The ability of latissimus dorsi transfer to improve elbow function has revealed encouraging results [6]. We report a clinical case with satisfying elbow and shoulder function after performed bipolar latissimus dorsi muscle transfer. This case report has been reported in line with the SCARE criteria [7].

## 2 Case presentation

A 26-year-old man was admitted to the outpatient clinic with a chief complaint of weakness in the left upper limb for 1.5 years following an accident. The patient motoric was M0 for shoulder abduction and elbow flexion and the range of motion for both movement is zero degree with Mayo Elbow Score (MES) of 60. He had normal wrist and hand function otherwise. Tingling sensations at the wrist and hand was present, while any sort of pain was denied by the patient. On physical examination, the patient's left arm showed signs of muscle atrophy, decreased sensibility from proximal arm to the hand, with limited ROM due to weakness. Neurological evaluation showed incomplete motoric function loss of the radial, median, and ulnar nerve. Sensoric evaluation revealed anesthesia at the Lateral side of the arm and forearm. Electromyographic evaluation revealed and confirmed the diagnosis of a incomplete left brachial plexopathy.

**Fig. 1:**
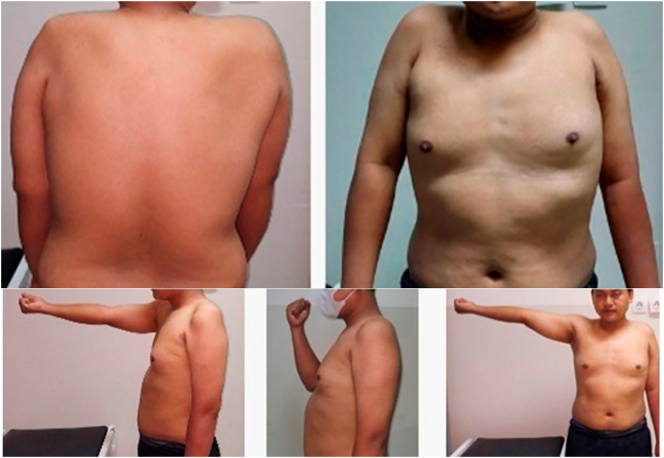
Physical examination showed no active movement of the left upper limb. Shoulder abduction, elbow flexion, and shoulder flexion test.

**Fig. 2:**
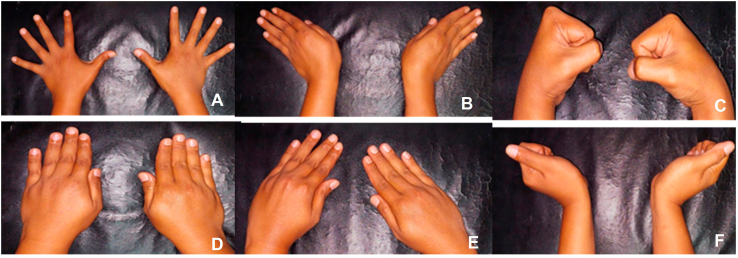
Physical examination of the arm showed normal motor function of the both hand: (A) hand abduction, (B) hand adduction, (c) wrist radial rotation, (d) wrist ulnar deviation, (e) thumb abduction, (f) wrist flexion.

**Fig. 3:**
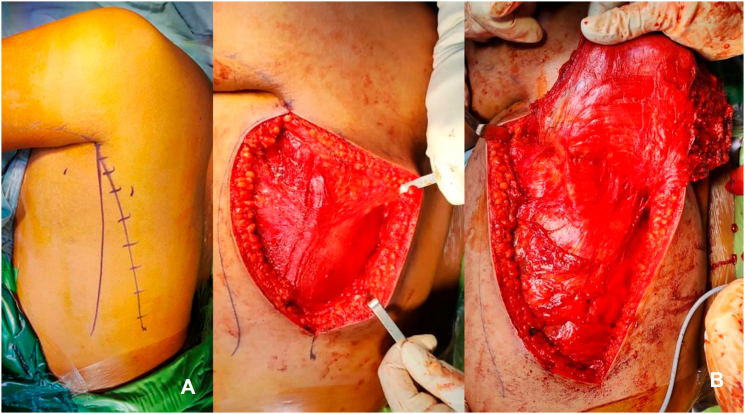
Latissimus dorsi muscle transfer. (A) Incision line draw, (B) identification of latissimus dorsi muscle.

**Fig. 4:**
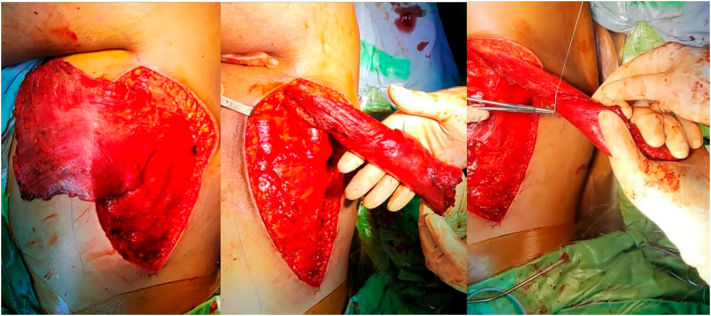
Latissimus dorsi muscle preparation.

The patient was unable to perform shoulder abduction and elbow flexion. After motoric examination, we observed good latissimus dorsi muscle strength. Prior to the operation, we referred the patient to a physical rehabilitation specialist to evaluate their motor strength through a manual muscle evaluation of the latissimus dorsi muscle. The minimum target strength is four. We performed needle EMG evaluation on latissimus dorsi muscle, which suggested an axonal lesion over the left superior and middle trunk that support the diagnosis of left brachial plexus injury. We decided to perform latissimus dorsi muscle transfer procedure for restoring elbow function. The latissimus dorsi muscle detached from its origin and insertion with the blood supply preserved, and a bipolar muscle transfer was performed. The proximal end was fixed to the AC joint and the distal end was sutured to the biceps tendon's insertion. Elbow flexion was restored immediately on the first day following the procedure accompanied by slight shoulder abduction. Six months later, the patient can perform 100 degree of elbow flexion, 30 degree of shoulder abduction with motoric strength of M4. Although he still had limited pronation and supination of the forearm, the MES increased to 80 (Fig. 1, Fig. 2, Fig. 3, Fig. 4).

Under general anesthesia, the surgery was successfully performed. The affected limb was elevated while in the lateral decubitus posture. The lateral border of the scapula and the posterior axillary fold were cut through the skin. The dissection proceeds laterally until the latissimus dorsi (LD) and teres major muscle bellies are inserted into the humerus. The plane between these muscles was discovered medially. The attachment site of the LD tendon is better exposed when the arm is rotated internally. Two sutures were placed on the muscle's surface before the latissimus dorsi tendon detached. This space was kept during the tensioning and reattachment of the augmented tendon. The sutures were spaced apart by 5 cm. The tendon was then detached from the humerus, and the latissimus muscle was freed medially using a pedicle musle transfer technique and dissected off its scapular origin to increase its excursion while protecting its neurovascular bundle.

**Fig. 5:**
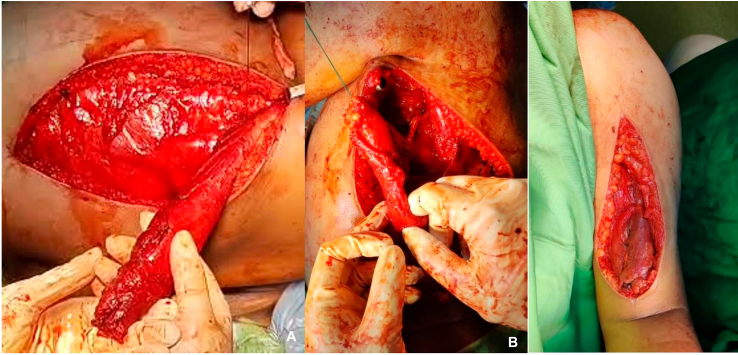
Latissimus dorsi muscle transfer. (A) Muscle detachment. (B) Post muscle transfer.

The length of the latissimus dorsi tendon was increased by harvesting an ipsilateral semitendinosus (ST) tendon graft. Ethibond No. 5 sutures were used to sew this in a fish-mouth end-to-end fashion, and the repair site was tensioned to take up any loose ends. The stretched tendon was then transferred superficially to the rotator cuff, beneath the deltoid and the posterior aspect of the acromion. The tendon graft was extracted via a second skin incision made above the middle trapezius, directly medial to the lateral aspect of the acromion, following its passage through the spinoglenoid notch (Fig. 5).

The lateral part of the acromion was covered by a subcutaneous tunnel without causing any damage to its periosteum or the soft tissue confluence where the denervated deltoid and trapezius attachments originate. A third skin incision was created across from the deltoid insertion in the humerus, and the tendon graft was then transmitted past the acromion in this tunnel to be recovered. Using a 4.5 mm drill bit, a bone tunnel was created in the humerus at the location of the deltoid insertion, and the tendon transplant was passed through the tunnel. Using ethibond no. 5 sutures, the recovered end of the graft was woven back into the main tendon and fastened in a 90° abduction of the shoulder.

**Fig. 6:**
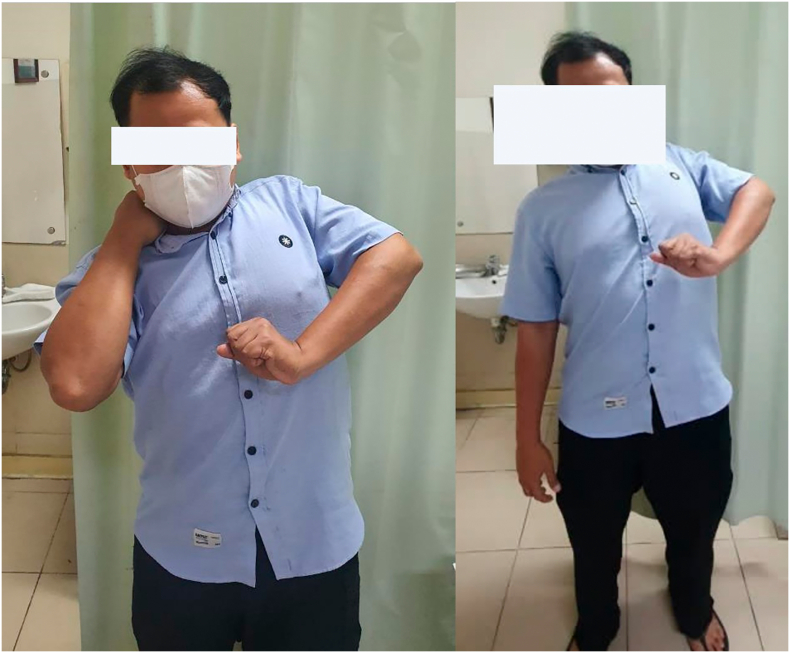
Follow up at 1 month after latissimus dorsi muscle transfer.

Post operatively, the arm was splinted with the elbow flexed to 90^0^, starting from full time splinting for the first three months, following overnight splinting for the next six months. Active flexion and extension were begun at six weeks, but passive extension was avoided for the first three months (Fig. 6).

At 6-months follow up after muscle transfer surgery from pectoralis major to biceps muscle, the patient reported improvement in upper limb movement with minimal pain at the surgical site. Numbness were present along the elbow to the fingers. The use of left upper extremity for daily activity was still limited. On physical examination, muscle atrophy was observed at the left pectoralis mayor, deltoid, bicep, tricep, brachialis and coracobrachialis muscle. The upper arm was hyperpigmented. Manual Muscle Test for shoulder adduction and abduction, shoulder flexion and extension scored 5/4, while elbow flexion scored 5/2. The patient was educated to continue physiotherapy and home exercises.

**Fig. 7:**
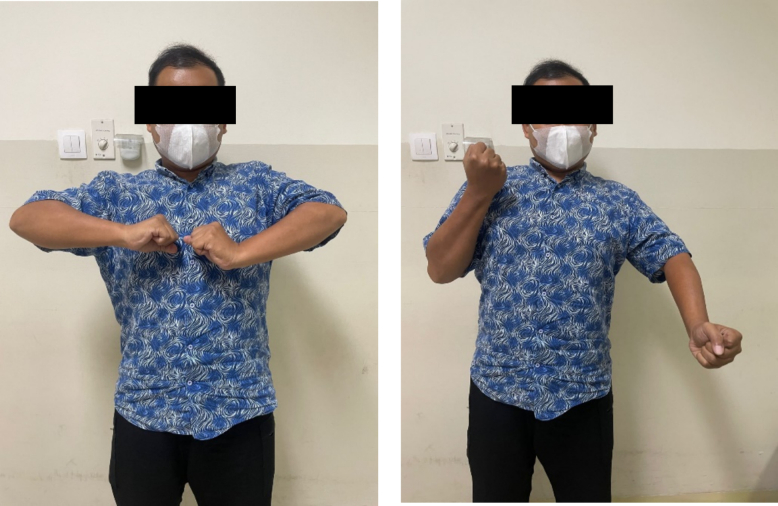
Follow up at 6 months after latissimus dorsi muscle transfer.

The circumference of arm is 2 cm different larger at right site and around forearm is 1 cm larger at right site. Sensoric of axillary, musculocutaneous, radial, and median nerve are decreased about 20–30 %, but ulnar nerve is still good (Fig. 7).

## 3 Discussion

Following motor vehicle crashes, traumatized brachial plexus injuries can happen and are frequently a component of a complex polytrauma presentation. The glenohumeral joint fracture and dislocation are the most often occurring injuries, and many patients may require surgery. Due to their complexity, brachial plexus injuries (BPI) necessitate extensive, ongoing therapy. Patients frequently experience difficulties with daily living tasks and going back to work, particularly in jobs requiring manual labor [8,9].

Even though the wrist and hand still function normally, BPI patients who have lost all of their elbow flexors—the biceps brachii and brachialis—are extremely frustrated by their incapacity to carry out daily tasks like eating, shaving, combing, and most occupational and recreational activities. Both the surgeon and the desperate patients will be extremely satisfied with reconstructive techniques to restore elbow flexion [10].

Many inventive strategies have been devised for the restoration of elbow flexion. The injured nerve can be sutured or re-innervated from other motor nerves if repair is done within six months of the injury. But other operative procedures are required if surgery is not done within this time frame, if there is a serious myogenic lesion of the biceps brachii muscle involved, or if the paralyzed biceps brachii muscle does not recover after surgery in a sufficient amount of time. Many methods have been reported for restoration of elbow flexion—for example, Steindler flexorplasty, anterior transposition of the triceps tendon, transfer of the pectoralis major to the biceps, shoulder arthrodesis and sternocleidomastoid transfer with extension via a fascia lata graft, unipolar or bipolar transfer of the latissimus dorsi muscle, and free muscle grafts with motor nerve transfer [10]. The easiest and most reliable procedure among them is the Steindler flexorplasty, provided that the flexor pronator muscle group is available for transfer [11]. Pronation contracture of the forearm, flexion contracture of the wrist, and flexion contracture of the elbow joint have all been reported to occur commonly following Steindler flexorplasty [12].

On the other hand, it has been reported that latissimus dorsi muscle transfer to the biceps brachii provides satisfactory elbow flexion without severe contractures. Because of the extra overshortening of transplanted muscle, we did not see any flexion contracture in this patient who had elbow flexion restored. Forearm pronation or supination contracture was not observed. Therefore, we propose that the first line of treatment should be the transfer of the latissimus dorsi muscle when its strength is normal. With paralysis of the latissimus dorsi muscle, the Steindler procedure should be selected when the power of the forearm flexor muscle is normal [13]. Because of its earlier return to function and bigger donor size, Vekris et al. also chose the pedicled latissimus dorsi transfer for elbow flexion. The patient was scheduled to undergo a bipolar muscle transfer utilizing the pedicle muscle transfer technique, with single point fixation applied to the ulnar tendon. This procedure aims to restore the function of arm supination, as the patient's pronation function is already adequate. Furthermore, compared to flexorplasty, it offers greater functional elbow motion and lift strength [5].

Because of the postganglionic lesion and the upper-type injury observed in this patient, the latissimus dorsi muscle, which receives innervation from the lower brachial plexus, remains a viable option for use (intraplexus transfer), minimizing donor muscle morbidity. In contrast, utilizing the trapezius muscle for transfer could impair scapulothoracic movement due to the resulting functional loss and detachment from its vasculature. The latissimus dorsi is a very dependable microvascular transfer that may be raised without difficulty. The muscle has a larger cross-sectional area than the gracilis and thus has the potential for a greater force of contraction. However, the anatomy of the muscle is such that it does not lend itself as well to being inserted into the upper arm. There is a short tendon of insertion and a broad musculoaponeurotic origin. The latissimus dorsi free vascularized flap also has the benefit of having a single motor nerve, a sizable vascular pedicle of 2–3 mm in diameter and 8–12 cm in length, and a significant skin island that can be harvested along with the muscle [8]. Because of the arm's relatively small subcutaneous pocket, several authors always prefer to take the muscle with some skin covering it. This helps to prevent applying too much pressure to the transferred muscle [14].

After the muscle tailoring, care should be given to ensure that the surplus skin is positioned on the anterior edge of the latissimus to face forward. The latissimus dorsi's posterior muscle fibers are reported to be shorter than its anterior counterparts. Therefore, all muscle fibers should be attached both cranially and caudally during the muscle tailoring process. Compared to Steindler flexorplasty, limitations include a longer operating duration and more thorough surgical dissection [6]. Results of arthroscopically assisted latissimus transfer have been superior to those of the conventional two-incision open approach. Arthroscopically assisted latissimus dorsi transfer for posterosuperior RCTs in 55 patients: reported outcomes. Patients' SSV increased from 26 % preoperatively to 71 % postoperatively, a comparable improvement to Gerber's findings. Furthermore, there were substantial enhancements in the degree of forward flexion, abduction, external rotation, and abduction strength.

In terms of latissimus dorsi muscle transfer surgery, outcomes may be advantageous if the transplanted muscle's tension is comparatively higher than the biceps brachii muscle's typical tension. The method of Zancolli and Mitre was used to determine the muscle tension; once both ends were sutured, the length of the transplanted muscle was modified to maintain the elbow at 100° of flexion and the forearm in total supination. Even after undergoing this process, a few individuals who had their elbow flexion restored need further muscle transfer shortening. After the procedure, the distal suture may become looser because it must be placed between the latissimus dorsi muscle belly and the stump of the biceps tendon [15].

In cases when the transferred muscle exhibits a strong contraction from the fully extended elbow, further shortening procedures might be necessary. If there is not a significant contraction from the fully extended elbow in the transferred muscle, then the additional shortening technique will not improve the muscular power. Furthermore, an elbow flexion contracture may result from overcorrection during the shortening process. The postoperative functional outcome was significantly impacted by the preoperative muscular power; optimal results require a latissimus dorsi muscle with normal strength prior to surgery. Since the latissimus dorsi contraction in this presenting patient was found to be relatively normal during preoperative testing, we decided not to undertake any additional shortening procedures [14]. In this presenting case we did not perform additional shortening procedure after preoperative assessment revealed relatively normal contraction from the latissimus dorsi. Complicated muscle transfer from the latissimus dorsi. Another instance in which, following latissimus transfer, the patient remains unable to flex the elbow. Further research is required to attain optimal muscle transfer tension.

## 4 Conclusion

After late brachial plexus injury, bipolar latissimus dorsi muscle flap yields an excellent result in elbow reconstruction. A successful postoperative outcome will be predicted by the preoperative assessment of donor muscle strength.

## Author contribution

Robiady, YS - Surgeon, study design, collecting data, analysis data, writing, submitting.

Malik, R - Surgeon, collecting data, analysis data, writing.

Ichwan, AR - Surgeon, collecting data, analysis data, writing.

## Consent

Written consent was obtained from the patient for publication of this case report and accompanying images. A copy of the written consent is available for review by the Editor-in-Chief of this journal on request.

## Consent for publication

Not applicable.

## Ethical approval

We certify this kind of manuscript does not require ethical approval (exemption) by the Ethical Committee of our institution.

## Guarantor

Realita Malik.

## Research registration number

Not applicable.

## Funding

This research did not receive any specific grant from funding agencies in the public, commercial, or not-for-profit sectors.

## Conflict of interest statement

The authors declare that they have no competing interests.

## Data Availability

Not applicable.
